# *Egln1^Tie2Cre^* Mice Exhibit Similar Therapeutic Responses to Sildenafil, Ambrisentan, and Treprostinil as Pulmonary Arterial Hypertension (PAH) Patients, Supporting *Egln1^Tie2Cre^* Mice as a Useful PAH Model

**DOI:** 10.3390/ijms24032391

**Published:** 2023-01-25

**Authors:** Yi Peng, Jingbo Dai, You-Yang Zhao

**Affiliations:** 1 Program for Lung and Vascular Biology, and Section for Injury Repair and Regeneration Research, Stanley Manne Children’s Research Institute, Ann & Robert H. Lurie Children’s Hospital of Chicago, Chicago, IL 60611, USA; 2 Department of Pediatrics, Division of Critical Care, Northwestern University Feinberg School of Medicine, Chicago, IL 60611, USA; 3 Department of Pharmacology, Northwestern University Feinberg School of Medicine, Chicago, IL 60611, USA; 4 Department of Medicine, Division of Pulmonary and Critical Care Medicine, Northwestern University Feinberg School of Medicine, Chicago, IL 60611, USA; 5 Feinberg Cardiovascular and Renal Research Institute, Northwestern University Feinberg School of Medicine, Chicago, IL 60611, USA

**Keywords:** ambrisentan, Egln1, HIF prolyl hydroxylase, pulmonary vascular remodeling, pulmonary arterial hypertension, pulmonary arterial hypertension animal model, sildenafil, treprostinil

## Abstract

Pulmonary arterial hypertension (PAH) is a progressive and inevitably fatal disease characterized by the progressive increase of pulmonary vascular resistance and obliterative pulmonary vascular remodeling, which lead to right-sided heart failure and premature death. Many of the genetically modified mouse models do not develop severe PH and occlusive vascular remodeling. *Egln1^Tie2Cre^* mice with *Tie2Cre*-mediated deletion of Egln1, which encodes hypoxia-inducible factor (HIF) prolyl hydroxylase 2 (PHD2), is the only mouse model with severe PAH, progressive occlusive pulmonary vascular remodeling, and right-sided heart failure leading to 50–80% mortality from the age of 3–6 months, indicating that the *Egln1^Tie2Cre^* mice model is a long-sought-after murine PAH model. However, it is unknown if *Egln1^Tie2Cre^* mice respond to FDA-approved PAH drugs in a way similar to PAH patients. Here, we tested the therapeutic effects of the three vasodilators: sildenafil (targeting nitric oxide signaling), ambrisentan (endothelin receptor antagonist), and treprostinil (prostacyclin analog) on *Egln1^Tie2Cre^* mice. All of them attenuated right ventricular systolic pressure (RVSP) in *Egln1^Tie2Cre^* mice consistent with their role as vasodilators. However, these drugs have no beneficial effects on pulmonary arterial function. Cardiac output was also markedly improved in *Egln1^Tie2Cre^* mice by any of the drug treatments. They only partially improved RV function and reduced RV hypertrophy and pulmonary vascular remodeling as well as improving short-term survival in a drug-dependent manner. These data demonstrate that *Egln1^Tie2Cre^* mice exhibit similar responses to these drugs as PAH patients seen in clinical trials. Thus, our study provides further evidence that the *Egln1^Tie2Cre^* mouse model of severe PAH is an ideal model of PAH and is potentially useful for enabling identification of drug targets and preclinical testing of novel PAH drug candidates.

## 1. Introduction

Pulmonary arterial hypertension (PAH) is a progressive and inevitably fatal disease resulting from various causes [1]. It is defined by a resting mean pulmonary artery pressure (PAP) greater than 20 mmHg and characterized by excessive vasoconstriction and obliterative pulmonary vascular remodeling which eventually cause right heart failure (RHF) and premature death [1,2,3]. The survival rate is approximately 50% in 5 years. Currently, five classes of drugs, endothelin receptor antagonists, phosphodiesterase-5 (PDE5) inhibitors, soluble guanylate cyclase (sGC) activators, prostacyclin analogues, and prostacyclin receptors agonists are approved by the United States Food and Drug Administration (FDA) to treat PAH [4]. These treatments mainly target the vasoconstriction abnormalities though the prostacyclin, nitric oxide (NO), and endothelin signaling pathways, but not the obliterative vascular remodeling abnormalities. Hence, the initial efficacy of PAH-targeted treatments has not always been maintained, only modest improvements are achieved in PAH patients’ morbidity and mortality [5].

Experimental animal models of PAH have been developed for preclinical testing of new pharmacotherapies including rats challenged with monocrotaline (MCT) [6] or chronic hypoxia plus administration of an inhibitor of vascular endothelial growth factor receptors (VEGFR), Sugen 5416 (SuHx) [7]. Although these rat models exhibit severe pulmonary vascular remodeling, they only partially replicate the clinical features of PAH with unstable PAH [8,9], which is in contrast to the progressive nature of clinical PAH. These treatments such as SuHx or hypoxia alone fail to induce severe PH with stable vascular remodeling in mice. Furthermore, various mouse models with genetic modifications have been generated [10,11] to delineate the molecular mechanisms responsible for pulmonary vascular remodeling but very few mouse models exhibit severe PH and occlusive vascular remodeling. Our previous studies have reported that *Tie2Cre*-mediated loss of hypoxia-inducible factor (HIF) prolyl hydroxylase 2 (PHD2, encoded by *Egln1*) in mice (*Egln1^Tie2Cre^*) induced spontaneously progressive severe PAH, which resembles all of the key pathological features of clinical PAH [12]. These clinical features include markedly elevated right ventricular systolic pressure (RVSP) ranging from 70–100 mmHg, severe RV hypertrophy and dysfunction, obliterative pulmonary vascular remodeling (including the formation of plexiform-like lesions), and 50% and 80% mortality at 3.5 and 6 months of age, respectively, with right heart failure (RHF). To further test if the *Egln1^Tie2Cre^* mouse model is an ideal preclinical PAH model, we determined the therapeutic responses of *Egln1^Tie2^* mice to three FDA-approved PAH drugs which are vasodilators representing classical therapies of PAH by targeting three distinct signaling pathways, prostacyclin analogs (treprostinil), endothelin receptor antagonists (ambrisentan), and PDE5 inhibitor (sildenafil). The current study demonstrates that treatment of *Egln1^Tie2Cre^* mice with these vasodilators largely replicates the clinical benefits of these drugs seen in PAH patients including short-term survival but with a lack of a long-term survival benefit, and partial improvement of pulmonary hemodynamics. This study provides further evidence that the *Egln1^Tie2Cre^* mouse model is an ideal preclinical model of clinical PAH. 

## 2. Results

### 2.1. Effects of Sildenafil, Ambrisentan, and Treprostinil on the Survival of Egln1^Tie2Cre^ Mice

**Figure 1:**
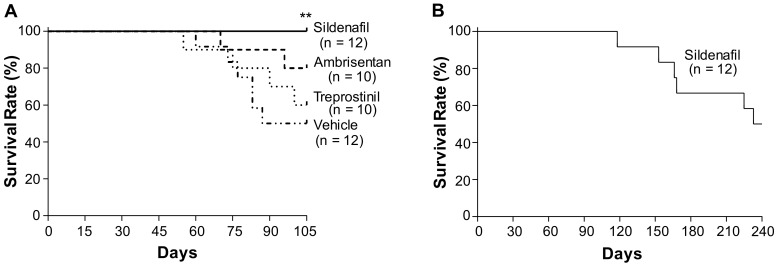
**Effects of chronic treatment of either sildenafil, ambrisentan, or treprostinil on the survival rate of *Egln1^Tie2Cre^* mice.** (**A**) At the age of 35 days, separate cohorts of *Egln1^Tie2Cre^* mice were treated with either a vehicle (PBS, oral daily), sildenafil (100 mg/kg, oral, daily), ambrisentan (10 mg/kg, oral, daily), or treprostinil (110 ng/kg/min, osmotic pump) until the age of 105 days. The survival rates were recorded. (**B**) The sildenafil-treated *Egln1^Tie2Cre^* mice were treated with sildenafil until the age of 240 days. The survival rates were recorded during this period. ** *p* < 0.01. log-rank (Mantel–Cox) test.

To assess the effects of FDA-approved vasodilators on *Egln1^Tie2Cre^* mice with established severe PAH [12,13,14], we treated 5-week-old mice with either sildenafil (100 mg/kg, oral, daily) [15], ambrisentan (10 mg/kg, oral, daily) [16], or treprostinil (110 ng/kg/min, subcutaneous infusion) [17]. The doses of sildenafil, ambrisentan, and treprostinil were selected based on previous studies that investigated the effects of these drugs in preclinical animal models and that demonstrated amelioration of established PAH in different rodent models. sildenafil treatment resulted in a markedly improved survival rate in *Egln1^Tie2Cre^* mice; all of the treated mice survived at the end of the 15-week study (i.e., 10-week treatment), whereas 50% of the vehicle-treated mice had died within this period (Figure 1A). Long-term treatment of sildenafil (i.e., 206-day treatment) showed a 50% survival rate (Figure 1B), consistent with the clinical observation that sildenafil is less effective in improving the long-term survival of PAH patients [18]. Ten-week treatment of ambrisentan or treprostinil resulted in survival rates of 80% and 60%, respectively, without statistical significance compared to a 50% survival rate for the vehicle-treated mice (Figure 1A).

### 2.2. Sildenafil, Ambrisentan, or Treprostinil Treatment Attenuated Right Ventricular Systolic Pressure (RVSP) with Neglectable Effects on Pulmonary Artery (PA) Function in Egln1^Tie2Cre^ Mice

**Figure 2:**
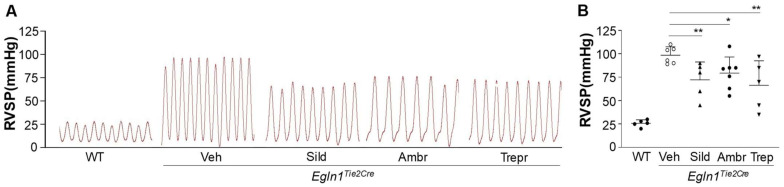
**Effects of chronic treatment of either sildenafil, ambrisentan, or treprostinil on pulmonary arterial hemodynamics in *Egln1^Tie2Cre^* mice.** (**A**) Representative RVSP tracings (1 s). *Egln1^Tie2Cre^* mice at the age of 35 days were treated with either a vehicle (PBS, oral, daily), sildenafil (100 mg/kg, oral, daily), ambrisentan (10 mg/kg, oral, daily), or treprostinil (110 ng/kg/min, osmotic pump) until age of 105 days (3.5 months). The RVSP was measured through jugular vein cannulation. (**B**) The RVSP measurement demonstrated a marked decrease in RVSP by either one of the three drugs compared to the vehicle-treated *Egln1^Tie2Cre^* mice. Data are expressed as mean ± SD (*n* = 5–8). WT = wild-type, Veh = vehicle, Sild = sildenafil, Ambr = ambrisentan, Trep = treprostinil. * *p* < 0.05; ** *p* < 0.01. One-way ANOVA with Dunnett’s post-hoc analysis.

**Figure 3:**
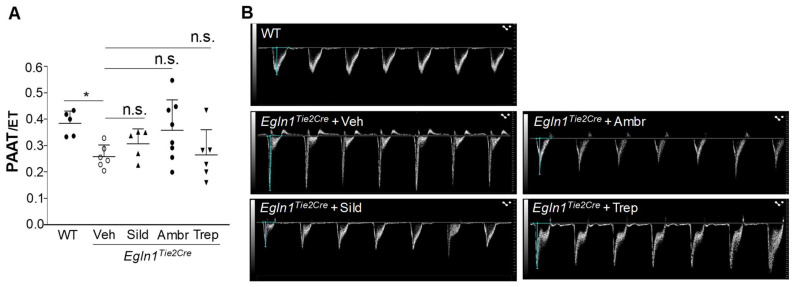
**Treatment with FDA-approved drugs had negligible effects on PA function.** (**A**) *Egln1^Tie2Cre^* mice at the age of 35 days were treated with either a vehicle (Veh), sildenafil (Sild) (100 mg/kg, oral, daily), ambrisentan (Ambr) (10 mg/kg, oral, daily), or treprostinil (Trep) (110 ng/kg/min, osmotic pump) until the age of 105 days. Echocardiography was carried out to determine PA function indicated by the PA AT/ET ratio. Data are expressed as mean ± SD (*n* = 5–8). (**B**) Representative micrographs of pulsed wave doppler of pulmonary arteries of WT mice and the vehicle- or drug-treated *Egln1^Tie2Cre^* mice. * *p* < 0.05. One-way ANOVA with Dunnett’s post-hoc analysis. n.s. = not significant.

To assess the effects of these vasodilators on PH, RVSP was measured. At the end of the 10-week treatment, all of the treatments with either of the drugs resulted in a partial but significant reduction in RVSP (Figure 2). However, echocardiography analysis of PA function indicated by the ratio of PA acceleration time/ejection time (PA AT/ET) showed that none of the drug treatments improved PA function (Figure 3).

### 2.3. The Effects of Sildenafil, Ambrisentan, or Treprostinil Treatment on RV Hypertrophy and Function in Egln1^Tie2Cre^ Mice

**Figure 4:**
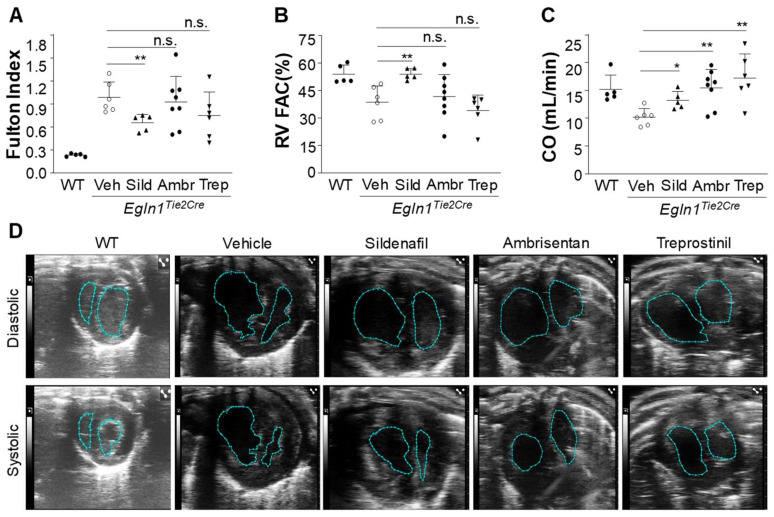
**Effects of treatment with either sildenafil, ambrisentan, or treprostinil on RV hypertrophy and function in *Egln1^Tie2Cre^* mice.** (**A**) Measurement of RV hypertrophy calculated by the RV/LV + S weight ratio. *Egln1^Tie2Cre^* mice at the age of 35 days were treated with either a vehicle (Veh, PBS), sildenafil (Sild) (100 mg/kg, oral, daily), ambrisentan (Ambr) (10 mg/kg, oral, daily), or treprostinil (Trep) (110 ng/kg/min, osmotic pump) until the age of 105 days. (**B**) Marked improvement in RV contractility by sildenafil but neither ambrisentan nor treprostinil treatment. Echocardiography was carried out to assess RV function by determination of RV fraction area change (RV FAC). (**C**) Marked improvement of cardiac output (CO) in *Egln1^Tie2Cre^* mice treated with either ambrisentan, treprostinil, or sildenafil. Cardiac output was calculated by LV tracing. (**D**) Representative echocardiography showing the RV and LV chamber area changes at systolic and diastolic. Data are expressed as mean ± SD (*n* = 5–8). * *p* < 0.05; ** *p* < 0.01. One-way ANOVA with Dunnett’s post-hoc analysis. n.s. = not significant.

In addition to hemodynamic improvement, sildenafil treatment also partially inhibited RV hypertrophy indicated by the RV/[(left ventricle + septum) (LV + S)] ratio, also called the Fulton index (Figure 4A). Neither ambrisentan nor treprostinil treatment had significant effects on RV hypertrophy (Figure 4A).

To assess the effects of these drugs on RV function and LV cardiac output, echocardiography was carried out at the end of the 10-week treatment. RV fraction area change (RV FAC) indicative of RV contractility was markedly improved in sildenafil-treated *Egln1^Tie2Cre^* mice (Figure 4B,D), ambrisentan and treprostinil treatment failed to improve RV contractility (Figure 4B,D). However, LV cardiac output in *Egln1^Tie2Cre^* mice treated with either sildenafil, ambrisentan, or treprostinil was markedly improved (Figure 4C,D). 

### 2.4. The Effects of Sildenafil, Ambrisentan, or Treprostinil Treatment on Obliterative Pulmonary Vascular Remodeling in Egln1^Tie2^ Mice

**Figure 5:**
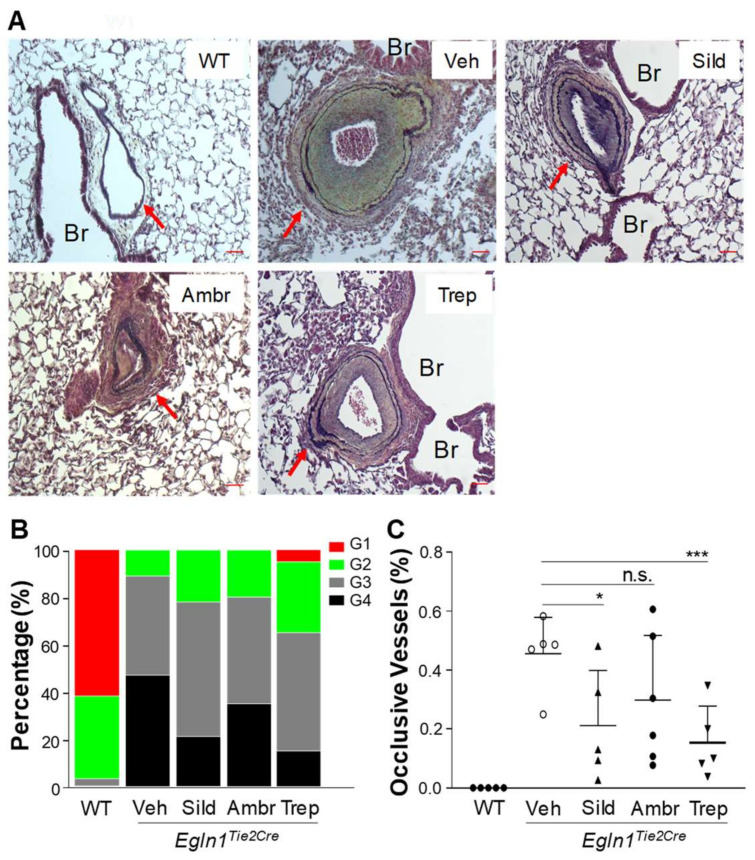
**Effects of treatment with either sildenafil, ambrisentan, or treprostinil on pulmonary vascular remodeling in *Egln1^Tie2Cre^* mice.** (**A**) Representative micrographs of Russell–Movat pentachrome staining of lung sections of WT and *Egln1^Tie2Cre^* mice treated with either a vehicle or an FDA-approved drug. *Egln1^Tie2Cre^* mice at the age of 35 days were treated with either a vehicle, sildenafil (100 mg/kg, oral, daily), ambrisentan (10 mg/kg, oral, daily), or treprostinil (110 ng/kg/min, osmotic pump) until the age of 105 days. Formalin-fixed lung sections were subjected to Russell–Movat pentachrome staining. Arrows point to vessels. Br = bronchiole. Scale bar, 50 µm. (**B**) Quantification of pulmonary vascular remodeling. Grade1 (G1), 2× vessel wall thickness/external diameter ratio <30%, indicating non-occlusion; G2, the wall/diameter ratio = 30–50%; G3, the wall/diameter ratio = 50–70% indicating partial occlusion; G4, the wall/diameter ratio ≥70% indicating occlusive lesions. *N* = 5 mice/group and 25–30 vessels/mouse. (**C**) Percentage of occlusive vessels. *N* = 5 mice/group and 25–30 vessels/mouse lung section were assessed for occlusive lesions (≥70% occlusion). * *p* < 0.05; *** *p* < 0.001. One-way ANOVA with Dunnett’s post-hoc analysis. n.s. = not significant.

Furthermore, we investigated the effects of sildenafil, ambrisentan, or treprostinil on pulmonary vascular remodeling in *Egln1^Tie2^* mice. At the age of 3.5 months with 10-week treatment of either drug or the vehicle, lung tissues were collected, formalin-fixed, and processed for paraffin sectioning and the sections were subjected to Russell–Movat pentachrome staining [12,13,19]. As shown previously [12,13,14,19], *Egln1^Tie2^* mice exhibited occlusive pulmonary vascular remodeling (Figure 5A). Quantification of occlusive vascular remodeling showed that either sildenafil or treprostinil treatment had caused a significant reduction in occlusive vessels compared to the vehicle group (Figure 5B,C). Ambrisentan treatment had minimal effect on occlusive pulmonary vascular remodeling (Figure 5B,C).

## 3. Discussion

**Figure 6:**
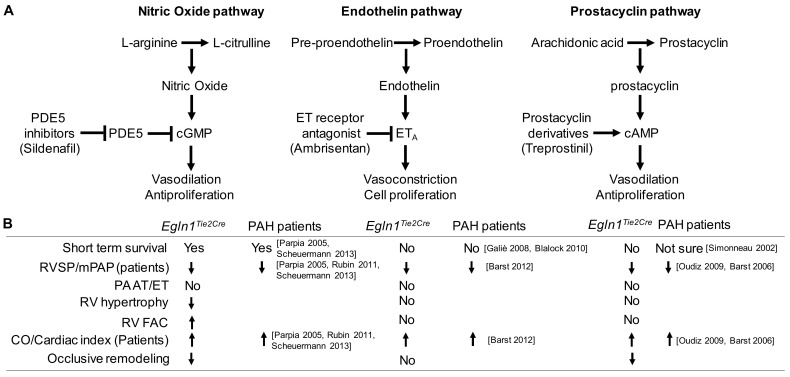
**Summary of the effects of the three drugs on *Egln1^Tie2Cre^* mice and PAH patients from clinical trials.** (**A**) Mechanisms of action of sildenafil, ambrisentan, and treprostinil. ET= endothelin-1. (**B**) The effects of sildenafil, ambrisentan, and treprostinil on survival and PAH phenotypes in *Egln1^Tie2Cre^* mice and PAH patients. mPAP = mean pulmonary arterial pressure. Paria 2005 [18]. Scheuemann 2013 [20], Rubin 2011 [21], Barst 2012 [22], Galiè 2008 [23], Blalock 2010 [24], Oudiz 2009 [25], Simonneau 2002 [26], Barst 2006 [27].

Our previous studies have reported that *Egln1^Tie2Cre^* mice closely resemble many key features of severe PAH seen in patients. Here, we have shown that *Egln1^Tie2Cre^* mice have similar therapeutic responses to the three classes of FDA-approved vasodilators as seen in clinical PAH. All three of the drugs can lower the RVSP in *Egln1^Tie2Cre^* mice consistent with their role as vasodilators and improve cardiac output (Figure 6). However, these drugs have only marginal effects on RV hypertrophy, RV function, pulmonary vascular remodeling, and short-term survival in a drug-dependent manner. None of the drugs have a significant effect on PA function. Together, these data further demonstrate that *Egln1^Tie2Cre^* mice appear to be the long-sought-after mouse model of clinical PAH.

*Tie2Cre*-mediated deletion of *Egln1* in ECs and hematopoietic cells spontaneously induces severe PAH in mice. Under basal conditions, *Egln1^Tie2Cre^* mice have progressive PAH with elevated RVSP (ranging from 70–100 mmHg) at the age of 3–4 months. These mice progressively die starting at the age of 1–2 months and exhibit 50% mortality by the age of 3.5 months, and 80% mortality by the age of 6 months. Severe RV hypertrophy (RV/LV + S ratio ranging from 0.75 to 1.1), a three-fold increase in RV wall thickness, RV dysfunction, and RV failure as well as PA dysfunction are observed in *Egln1^Tie2Cre^* mice [12,13,14,19,28]. More importantly, thickening of intima, media, and adventitia and neointima occlusion as well as the formation of plexiform-like lesions are prominent in pulmonary vessels from *Egln1^Tie2Cre^* mice at ages of older than 3 months [12,13,14,19]. All of the data demonstrate that *Egln1^Tie2Cre^* mice develop spontaneous progressive PAH with stable occlusive pulmonary vascular remodeling and RV failure leading to high mortality, as seen in patients with severe PAH. The profile of gene expression in the lungs of *Egln1^Tie2Cre^* mice indicates that many of the PAH-causing genes such as bone morphogenetic protein receptor type 2 (*BMPR2*), activin A-receptor-like type 1 (*ACVRL1*), endothelin-1 (EDN1), apelin (APLN), IL6, and caveolin 1 (*CAV1*) are altered, as seen in IPAH patients [12,29]. A recent study has also shown excessive oxidative/nitrative stress in *Egln1^Tie2Cre^* mice, another characteristic feature of clinical PAH [14]. Importantly, all of these PAH features of *Egln1^Tie2Cre^* mice are progressive and spontaneously irreversible. One possible limitation of this animal model is the development of PAH at its early age, for example at 3 weeks of age, which may be different from PAH patients. Except PAH in patients with genetic mutation(s), PAH is more prominent in adults and considered as an adult disease. As PAH is difficult to be diagnosed at the early stage, it is possible that many patients have mild PAH in younger life. *Egln1^Tie2Cre^* mice develop progressive PAH with much higher RVSP and more severe RV hypertrophy at 3–4 months of age compared to 3–5 weeks of age [12,13,14]. Occlusive pulmonary vascular remodeling is not evident until the age of 2 months and becomes prominent at the age of 3.5 months [12,13,14]. The adolescent signaling mediating PAH development may differ from what is observed in adults. However, *Egln1^Tie2^* mice exhibit altered expression of many genes involved in the pathogenesis of PAH. Among the 21 PAH-causing/associated genes analyzed, mRNA expression of 18 genes are dysregulated in *Egln1^Tie2Cre^* lungs [12]. Together, these data strongly support *Egln1^Tie2Cre^* mice as an ideal murine model of PAH.

The limitation of animal PH models is the translational gap for many drug candidates which were promising during preclinical evaluation but had limited success during subsequent clinical trials. The MCT-rats have marked elevation of RVSP, RV dysfunction, and moderate mortality at 4–6 weeks after administration, but a lack of plexiform lesions, the typical manifestation of severe PAH. Moreover, the response to MCT is variable among strains and even animals because of differences in hepatic metabolism by cytochrome P-450 [30]. PH induced by low doses of MCT is reversible [8]. The SuHx-rat is also a partially reversible model of PH which exhibits low mortality rates compared to human PAH although it develops occlusive pulmonary vascular remodeling [8]. Hypoxia- or SuHx-induced PH in mice is in general mild without occlusive pulmonary vascular remodeling and also reversible [31]. These flaws of experimental PH models may explain why PAH-targeting treatments have limited success in clinical trials of PAH patients. In comparison with the commonly used MCT-rat and SuHx-rat PH models, the *Egln1^Tie2Cre^* mouse model resembles many of the key pathological features of clinical PAH, with progressive and stable occlusive pulmonary vascular remodeling. Moreover, the *Egln1^Tie2Cre^* mice generally cost less to maintain, and the tools for genetic manipulation are more feasible [32]. Altogether, *Egln1^Tie2Cre^* mice may be a more ideal preclinical PAH model. Employing this model, our previous study tested the therapeutic effects of compound 76 (C76) [20] which selectively inhibits hypoxia-inducible factor 2A (HIF2α) translation. C76 treatment markedly inhibited PAH progression and occlusive pulmonary vascular remodeling and promoted the survival of *Egln1^Tie2^* mice indicating that inhibition of HIF-2α is a novel effective therapeutic strategy for PAH in patients [13]. It is with great interest to see whether the HIF-2α inhibitor drug Welireg, recently approved for treatment of adult patients with von Hippel–Lindau disease who require therapy for associated renal cell carcinoma, is effective in inhibiting PAH progression and occlusive pulmonary vascular remodeling and improving survival of PAH patients.

In this study, we have shown that current FDA-approved vasodilating drugs including treprostinil, ambrisentan, and sildenafil can partially attenuate RVSP without improvement in PA function in *Egln1^Tie2Cre^* mice, which is consistent with their vasodilating effects, and also improve cardiac output. These drugs have been shown to reduce mean pulmonary artery pressure and the improve cardiac index which is normalized cardiac output by body surface area in various PAH clinical trials (Figure 6) [18,21,22,23,24,25,26,27,33,34,35]. However, only sildenafil treatment promotes the short-term survival of *Egln1^Tie2Cre^* mice, but there is still 50% mortality at the age of 8 months after about 7 months of treatment. Clinical studies show that sildenafil significantly improved the survival rate, 68% to 93% in one year, but it was less effective for 3-year survival; the incidence of clinical worsening was not different between sildenafil-treated and placebo-treated PAH patients [18,36]. Besides improvement of RVSP and cardiac output, sildenafil-treated *Egln1^Tie2^* mice at the age of 3.5 months also exhibited reduced RV hypertrophy, improved RV contractility and reduced occlusive vascular remodeling. Together, these improvements may explain the unique beneficial effects of sildenafil treatment on short-term survival. Other preclinical studies have also shown that sildenafil treatment inhibits increases in RVSP and RV hypertrophy and attenuates pulmonary vascular remodeling [37,38].

Our studies have shown that, overall, sildenafil showed better therapeutic effects in our mouse model compared to ambrisentan and treprostinil. Although ambrisentan and treprostinil improved cardiac output, neither ambrisentan nor treprostinil treatment resulted in reduced RV hypertrophy and improved RV FAC, indicative of RV contractility. RV FAC is a measurement that provides an estimate of global RV systolic function, and it does not always reflect cardiac output since LV also contributes to the overall cardiac output. The elevated cardiac output may be a result of lower PVR in these vasodilator treatment groups independent of RV systolic function.

Similar to sildenafil, treprostinil treatment also attenuated occlusive pulmonary vascular remodeling. A previous study reported that treprostinil treatment can target the DP1 receptor that in part blocked the progression of hypoxia-induced PH in mice and attenuated pulmonary vascular remodeling, indicating that treprostinil may target different signals other than a vasodilator [39]. Among the three drugs, only ambrisentan has a negligible effect on pulmonary vascular remodeling. Ambrisentan-treated *Egln1^Tie2Cre^* mice exhibit a similar degree of occlusive vascular remodeling as the vehicle-treated mice. Ambrisentan is an endothelin-1-receptor-A-(ET_A_)-selective antagonist. Inhibition of ET_A_ may not be sufficient to inhibit cell proliferation and thus has minimal effects on pulmonary vascular remodeling. It has been shown that decreased RV hypertrophy and enhanced survival are seen only with the dual ET_A_ and ET_B_ antagonist, suggesting that inhibition of both ET_A_/ET_B_ is required to have maximal beneficial effects [40]. Clinical studies have shown that short-term ambrisentan treatment improves 6-minute walk distance (6MWD) and cardiopulmonary hemodynamics at 12 weeks [23,34]. However, the beneficial effects of ambrisentan on PVR and overall survival may require long-term treatment (>1 year) [24,25]. Our study shows a trend of improvement of short-term survival in ambrisentan-treated *Egln1^Tie2Cre^* mice. Future studies are warranted to determine if long-term ambrisentan treatment can markedly improve the survival of *Egln1^Tie2Cre^* mice.

There are some limitations of this study. Although the study shows that sildenafil treatment delayed mortality, the temporal progression of cardiovascular function was not assessed. Future studies are warranted to assess the echocardiographic phenotype at various intervals (e.g., every 30 days) up to 240 days to determine whether the mortality by 240 days is ascribed to progressive deterioration of the cardiovascular phenotype such as right ventricular contractility indicated by RV FAC over time and the underlying mechanisms of improved survival by sildenafil treatment. Based on the 3 Rs (replacement, reduction, and refinement) for the more ethical use of animals, the study used only one PBS vehicle control group. The lack of an osmotic pump vehicle control group in the treprostinil treatment study is another limitation. Given that the osmotic pump was implanted in the back subcutaneously, minor surgery should not affect the pulmonary hypertensive phenotypes including cardiac function and mortality. Subcutaneous treatment of treprostinil by the pump has been used in PAH patients, which further supports that the pump’s use itself does not affect pulmonary hypertensive phenotypes. Thus, we believe that the osmotic pump vehicle control should be similar to the PBS vehicle control.

In conclusion, the current study assessed the efficacy of three FDA-approved vasodilating drugs for the treatment of PAH in *Egln1^Tie2Cre^* mice who developed spontaneously progressive severe PAH with stable occlusive pulmonary vascular remodeling and RV failure. Although all of them can attenuate RVSP consistent with their role as vasodilators and improved cardiac output, these drugs have no beneficial effects on PA function and only partially improve RV function, RV hypertrophy, and pulmonary vascular remodeling as well as survival effects in a drug-dependent manner. These data replicate the clinical benefits of these drugs in multiple clinical trials on PAH patients. Thus, our study provides further evidence that the *Egln1^Tie2Cre^* mouse model of severe PAH is an ideal model of clinical PAH and potentially useful for enabling identification of valuable, druggable targets and preclinical testing of novel PAH drug candidates.

## 4. Materials and Methods

Mice. *Egln1^Tie2Cre^* mice were generated by breeding *Egln1* floxed mice (strain #009672, The Jackson Laboratory) into the genetic background of *Tie2Cre* mice (strain #008863, The Jackson Laboratory). Both are of a C57BL/6J background. Both male and female *Egln1^Tie2Cre^* mice at the age of 5 weeks were randomly assigned for treatment with either a vehicle (PBS), sildenafil (100 mg/kg, oral gavage, daily), ambrisentan (10 mg/kg, oral gavage, daily), or treprostinil (110 ng/kg/min, subcutaneous infusion) for 10 weeks. sildenafil treatment was maintained for 206 days for monitoring long-term survival. All of the experiments were performed according to NIH guidelines on the use of laboratory animals. The animal care and study protocols were approved by the institutional Animal Care and Use Committee of Northwestern University Feinberg School of Medicine.

### 4.1. Surgical Procedures for Osmotic Minipump Implantation in Mice

The osmotic minipumps (model number 1004; infusion rate 0.1 uL·min^−1^; Alzet, CA, USA) were filled with treprostinil (Remodulin; United Therapeutics Corp., Research Triangle, NC, USA) and equilibrated at 37 °C in PBS for 48 h before implantation. The mice were anaesthetized with 3% isoflurane. The minipump was implanted subcutaneously on the back. The incisions were closed with surgical suture. The animals received post-operative analgesia with buprenorphine injections twice.

### 4.2. Echocardiography

Echocardiography was performed with a vevo3100 ultrasound machine (FujiFilm Visual sonic Inc., Toronto, ON, Canada) in the Preclinical Animal Models Core at Northwestern University Feinberg School of Medicine as described previously [12,13,19]. Briefly, the mice were anesthetized using 1–1.5% isoflurane via a face mask and subjected to transthoracic echocardiography using transducers MS-550D, 22–55 MHz. The ight ventricles and left ventricles were measured by parasternal short-axis views at the mid-papillary level in B-mode and M-mode echocardiograms. Cardiac output was measured from the left ventricle. Pulse-wave Doppler echocardiography mode was used to record the pulmonary artery blood outflow in the short-axis view to measure PA, AT, and ET.

### 4.3. RV Hemodynamic Measurements

RVSP was measured with a 1.4 F pressure transducer catheter (Millar Instruments) as described previously [12,13,19].

### 4.4. Histological Assessment of Pulmonary Vascular Remodeling

The mouse lungs were fixed for 5 min by instillation of 10% PBS-buffered formalin through tracheal catheterization at a transpulmonary pressure of 15 cm H_2_O and then overnight at 4 °C with agitation. After paraffin processing, the tissues were cut into semi-thin 5 μm-thick sections. The mouse lung sections were then dewaxed, dehydrated, and stained with a Russell–Movat pentachrome staining kit (American MasterTech). The sections were then imaged with an APerio CS2 light microscope (Leica Biosystems). The vessels were viewed under a light microscope with a 20× objective in at least 25 nonoverlapping fields of each section (12). Pulmonary vascular remodeling was assessed based on the ratio of 2× vessel wall thickness divided by the external wall diameter. Grade 1 (G1), the wall/diameter ratio <30%, indicating non-occlusion; G2, the wall/diameter ratio = 30–50%; G3, the wall/diameter ratio = 50–70%, indicating partial occlusion; G4, the wall/diameter ratio ≥70%, indicating occlusive lesions. The percentage of occlusive vessels was calculated by the number of G4 vessels divided by the total number of vessels quantified in each mouse section.

### 4.5. Statistical Analysis

Statistical analysis of the data was carried out with GraphPad Prism 7 (GraphPad Software, Inc.). The data distribution was assessed by the Shapiro–Wilk test. All of the data except WT cardiac output data passed the normal distribution test. One-way ANOVA with Dunnett’s post-hoc analysis was used for multiple group comparison. Survival analysis was determined by the log-rank (Mantel–Cox) test. *p*-values of <0.05 were considered significant. Data are expressed as mean ± SD. Bars in dot plot figures represent the mean.

## Data Availability

All the data were published in this article.
